# The effects of rTMS combined with blood flow restriction low-intensity resistance training on skeletal muscle mass, strength, and physical function in postmenopausal women: a single-blind randomized controlled trial protocol

**DOI:** 10.3389/fgwh.2025.1681138

**Published:** 2025-11-10

**Authors:** Xiangdi Dai, Yuxiang Wu, Yuan Yuan, Binhua Lu, Xiaowei Wang, Guodong Xu, Jie Zhuang

**Affiliations:** 1 School of Exercise and Health, Shanghai University of Sports, Shanghai, China; 2 School of Physical Education, Jianghan University, Wuhan, China; 3 Department of Radiology, Jianghan University Hospital, Wuhan, China

**Keywords:** skeletal muscle, menopausal women, randomized controlled trial, resistance training, blood flow restriction, transcranial magnetic stimulation

## Abstract

**Background:**

Skeletal muscle serves as the primary source of power for body movement. Due to the transition of menopause, older women experience a relatively faster decline in skeletal muscle mass and function, making them more susceptible to age-related skeletal muscle disorders such as sarcopenia, which can lead to adverse outcomes such as falls, fractures, disability, or even death. Blood flow restriction low-intensity resistance training (LI-BFRT) can effectively enhance muscle strength and promote skeletal muscle growth, while repetitive transcranial magnetic stimulation (rTMS) has previously been shown to improve motor cortex excitability and limb motor function. We describe a trial protocol to investigate the effects of a 12-week intervention combining rTMS with LI-BFRT on skeletal muscle mass and physical function in community-dwelling postmenopausal women.

**Methods:**

This single-blind, randomized controlled trial will recruit 54 eligible community-dwelling postmenopausal women aged 50–65 years who have been naturally postmenopausal for 1 year or more. Participants will be randomly assigned in a 1:1:1 ratio to the rTMS combined with the blood flow restriction low-intensity resistance training group (rTMS + LI-BFRT), the blood flow restriction low-intensity resistance training group (LI-BFRT), and the control group, respectively, for a 12-week intervention period. Participants will undergo assessments at baseline (Week 0), immediately after the intervention (Week 12), and long-term follow-up (Week 24). The primary outcomes include lower limb muscle mass, muscle strength in both the upper and lower limbs; secondary outcomes include body composition, physical function (5-time sit-to-stand test, 30-second stand test, timed up-and-go test, 30-second arm curl test), motor cortex excitability, and clinical blood markers related to neural and muscular function.

**Discussion:**

We have combined central and peripheral motor activation methods for the first time, attempting to use BFRT in combination with rTMS intervention to recruit more motor units by increasing motor cortex excitability, thereby enhancing skeletal muscle motor ability under BFRT and examining the intervention effects on skeletal muscle mass and strength in postmenopausal women. This will provide a new paradigm for healthy intervention in the skeletal muscles of postmenopausal women.

**Clinical Trial Registration:**

http://www.chictr.org.cn, Chinese Clinical Trial Registry ChiCTR2400086697.

## 1 Introduction

Skeletal muscle is the fundamental driving force for maintaining muscle tone, preserving body posture, and accomplishing various physical activities, while also playing a crucial role in metabolism (1).With advancing age, the physical functions of middle-aged and elderly individuals gradually decline, including skeletal muscle mass and strength (2).This decline in muscle function and physical activity capacity appears more pronounced in older women. During the aging process, women inevitably experience the menopausal transition, leading to abrupt changes in hormone levels, particularly estradiol (3, 4). Estradiol is responsible for regulating the menstrual cycle and developing and maintaining female secondary sexual characteristics. It also promotes muscle health by enhancing muscle regeneration and stimulating the proliferation of muscle satellite cells (5, 6). Therefore, this hormonal shift is believed to impact the entire body, especially skeletal muscle composition, causing older women to accumulate more adipose tissue while gradually losing total muscle mass (7, 8). Previous studies have shown (9, 10) that postmenopausal women experience faster declines in muscle mass, muscle strength, and bone mineral density than age-matched men, making them more susceptible to chronic diseases such as osteoporosis and sarcopenia. As an age-related systemic and progressive skeletal muscle disorder, sarcopenia is characterized by generalized loss of muscle mass, reduced muscle strength, and impaired physical function (11). It is closely associated with adverse outcomes such as falls, fractures, physical disability, and even mortality (12, 13), emerging as one of the most common and critical diseases affecting mobility in older adults. Additionally, our previous research has found (14), a significant interaction between menopausal status and sarcopenia on cardiovascular disease (CVD) risk, where postmenopausal women with sarcopenia face a higher risk of CVD. Therefore, implementing proactive interventions for skeletal muscle in menopausal women to prevent or delay the onset of sarcopenia is of great medical and social significance for improving the health and quality of life in older women.

It is currently widely recognized that exercise intervention is a key measure for preventing and treating the decline in skeletal muscle mass and strength (15). Among these, resistance training is an effective method for preventing and treating the decline in skeletal muscle mass and strength, as it can enhance skeletal muscle strength through mechanisms such as regulating protein metabolic balance and reducing mitochondrial aging (16). Research has shown that high-intensity resistance training is more effective in alleviating sarcopenia in older people (17, 18), However, studies also point out that older people may not be able to tolerate the stress imposed by high-intensity resistance training on connective tissue, bones, and joints, which can lead to adverse reactions and increase the risk of injury (19), as a result, they often struggle to achieve the recommended exercise intensity and load (20). Therefore, new methods and approaches are needed to enhance the safety and adherence of exercise interventions for older people while achieving intervention effects comparable to those of high-intensity resistance training.

In recent years, blood flow restriction combined with low-intensity resistance training (LI-BFRT) has garnered increasing attention (21, 22). BFRT is a functional rehabilitation method that uses tourniquets or similar devices to apply pressure to the proximal end of the limbs, thereby restricting venous blood return to the distal end. This method effectively stimulates skeletal muscle growth, enhances skeletal muscle adaptability, improves muscle strength, inhibits muscle atrophy, improves blood circulation, and optimizes skeletal muscle capillary distribution, thereby promoting skeletal muscle functional rehabilitation (23, 24). Previous studies have confirmed that combining blood flow restriction with low-intensity resistance training (20%–30% of one-repetition maximum [1RM]) is an effective intervention for stimulating muscle fiber thickening and strength gains in older people, achieving effects similar to those of high-intensity resistance training (25), However, due to potential confounding factors associated with BFRT (such as gender and training volume) (19), further research is needed to provide evidence for the effective application of BFRT in older people (26). Although BFRT shows promising application potential, due to factors such as age and safety considerations, the blood flow restriction pressure and resistance load for postmenopausal women often cannot be set at higher levels, resulting in improvements in skeletal muscle mass and function, but these improvements still fall short of the effects achieved by high-intensity resistance training alone (27).

Research has shown that the increase in skeletal muscle strength during long-term training practice involves two aspects: adaptive hypertrophy of muscle fibers (peripheral factors) and an increase in the number of motor units recruited by the nervous system (central factors). In recent years, the causes of sarcopenia associated with age-related deterioration of nervous system function have attracted the attention of researchers (28, 29). During the normal aging process, the motor cortex undergoes significant morphological changes (30). Recent studies suggest that age-related diseases such as sarcopenia may be caused by cortical atrophy, reduced cortical output, motor neuron apoptosis, decreased action potential frequency, reduced recruitment of motor units, and alterations in neuromuscular junctions associated with neurons (31, 32).

Transcranial magnetic stimulation (TMS) is a magnetic stimulation technique based on the principles of electromagnetic induction and conversion. It employs time-varying magnetic fields to stimulate the cerebral cortex and peripheral nerves, inducing currents that alter the action potentials of neurons. This process influences neurotransmitter metabolism and neural electrical activity within the brain, thereby triggering a series of physiological and biochemical responses (33). It has been used in the treatment of neurological and psychiatric disorders associated with excitatory dysfunction of the brain, with rTMS commonly employed to modulate cortical excitability, as reflected by changes in motor-evoked potentials (MEPs). In interventions targeting post-stroke motor function, studies have found that rTMS can significantly alter motor cortex excitability and plasticity (34), optimize motor cortex plasticity changes induced by motor training, improve limb motor function (35, 36), and enhance the long-term effects of motor training in stroke patients. Currently, there are no reported applications of rTMS for interventions targeting individuals with reduced skeletal muscle mass and strength or sarcopenia, particularly postmenopausal women.

In summary, this study aims to investigate the effects of a 12-week LI-BFRT combined with rTMS intervention on skeletal muscle mass and strength, as well as physical function, in postmenopausal women. It will also quantitatively analyze changes in motor cortex excitability related to neuromuscular activity and hematological parameters before and after the exercise intervention, exploring potential mechanisms. Our findings will provide new insights and experimental evidence for effectively preventing or delaying the onset and progression of sarcopenia in older women.

## 2 Methods and analysis

### 2.1 Study design

**Figure 1:**
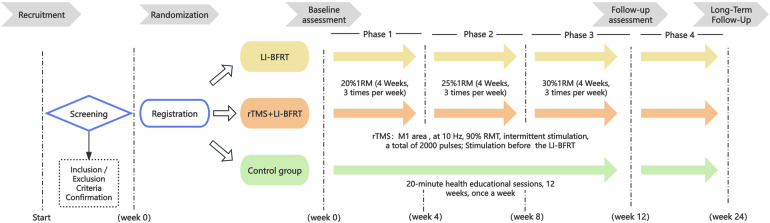
Flow chart showing the participant recruitment, intervention and assessment process. LI-BFRT, low intensity-blood flow restriction training; rTMS, repetitive transcranial magnetic stimulation; 1RM, 1 repetition maximum.

This is a 12-week, single-center, single-blind randomized controlled trial study. This study has been approved by the Ethics Committee of Shanghai University of Sport (Approval Number: 102772024RT078) and registered in the China Clinical Trial Registry (Registration Number: ChiCTR2400086697, registered on July 9, 2024). All participants provided written informed consent prior to enrollment. The study was conducted in accordance with the Declaration of Helsinki. The experimental flowchart is shown in Figure 1.

This study recruited healthy postmenopausal women. After applying inclusion and exclusion criteria, eligible participants were randomly assigned to the rTMS + LI-BFRT group, LI-BFRT group, and control group in a 1:1:1 ratio. Researchers confirmed the basic information of all participants who had signed informed consent forms (age, date of birth, contact information); after confirmation, blood samples were collected from participants, and height, weight, and body composition were measured. Subsequently, tests were conducted to assess skeletal muscle mass, skeletal muscle strength, motor cortex excitability, and motor performance. Participants in the two intervention groups received a 12-week supervised training intervention, three times per week. The control group was instructed to maintain their previous lifestyle and undergo a 12-week health education program, once per week. The program comprises weekly lectures on general health topics (nutrition, sleep hygiene, chronic disease awareness) and explicitly does not include active physical training or exercise prescriptions. All participants were assessed at three time points: pre-intervention (Week 0), post-intervention (Week 12), and at long-term follow-up (Week 24). The intervention and assessment schedule are outlined in Table 1.

**Table 1:** The schedule of participant recruitment, intervention, and study assessment.

Variables	Recruitment and screening	Baseline assessment	Intervention (Week 1-Week12)		Follow-up assessment	Long-term Follow-up assessment
Time-point		Week 0	Week 4	Week 8	Week 12	Week 24
Information consent	X					
Inclusion and exclusion criteria	X					
Demographic data	X					
Randomization	X					
AOP test		X				
RMT test		X				
Resistance training load		X	X	X		
Primary outcomes						
Thigh muscle CSA		X			X	X
Calf muscles CSA		X			X	X
Hand grip strength		X			X	X
Knee extension MVC		X			X	X
Secondary outcome						
Body composition		X			X	X
5-times chair stand test		X			X	X
30-Second Chair Stand test		X			X	X
Timed Up and Go test		X			X	X
30-Second Arm Curl Test		X			X	X
MEPs test		X			X	X
Hematological parameters		X			X	X

AOP, arterial occlusion pressure; RMT, resting motor threshold; muscle CSA, muscle cross-sectional area; MVC, maximal voluntary contraction; MEPs, motor-evoked potentials.

### 2.2 Inclusion criteria

1. Aged 50–65 years old
2. Naturally menopausal for ≥1year
3. Right-handed
4. Irregular exercisers (<3 times/week and 30 min/time in the past three months).

### 2.3 Exclusion criteria

1. History of major surgery or fracture within the previous 6 months
2. Contraindications to exercise, such as severe cardiovascular disease, severe osteoporosis, severe joint inflammation or diagnosed neurological disorders, including but not limited to: stroke, Parkinson's disease, epilepsy, and other neuromuscular diseases
3. Presence of metallic foreign bodies in the body
4. Inability to understand and complete the test, such as those with cognitive impairment, hearing impairment, or visual impairment

### 2.4 Randomization and blinding

Participants meeting the inclusion and exclusion criteria will be randomly assigned to the rTMS + LI-BFRT group, LI-BFRT group, and control group at a 1:1:1 ratio. The randomization sequence will be generated by an independent statistician using a computer program with block randomization to ensure balanced sample sizes across groups throughout the enrollment process. Group allocation results will be concealed in numbered, sealed, opaque envelopes, which are stored and distributed by researchers not involved in recruitment, intervention, or assessment to achieve allocation concealment. Outcome assessors and data analysts will remain blinded to participants' group assignments throughout the study. Researchers implementing the intervention are required to withhold group information during assessments.

### 2.5 Interventions

Pre-intervention procedures: Before the intervention, participants in the rTMS + LI-BFRT and LI-BFRT groups underwent a one-repetition maximum (1RM) test and arterial occlusion pressure (AOP) measurement, and were trained to use the 6–20 Borg Rating of Perceived Exertion (RPE) scale to determine target resistance loads. Exercise loads were applied using appropriately weighted dumbbells. Missed sessions were rescheduled within the same week to maintain consistent intervention volume. All participants attended a pre-intervention orientation to familiarize themselves with the procedures.

Adherence and fidelity: All sessions were supervised by trained researchers. Attendance and completion of prescribed exercise sets were recorded digitally. Any missed sessions or protocol deviations were documented. Adherence was calculated as the percentage of completed sessions relative to the total prescribed, with an attendance rate of ≥ 80% considered a valid indicator of intervention effectiveness. Detailed intervention protocols for all groups are provided in Table 2.

**Table 2:** Details of the three groups of protocol.

Weeks	Group	Specific intervention content	Sets	Repetitions	Interval rest time between sets	Intensity	Frequency
1–4	LI-BFRT	Seated biceps curl, Seated shoulder press Seated knee extension, Squat	4	12, 10, 10, 10	30s	Phase 1 20%1RM	3times/week
	rTMS + LI-BFRT	M1 area, at 10 Hz, 90%RMT, intermittent stimulation, a total of 2,000 pulses; LI-BFRT immediately after the stimulation ends.					
	CG	Education session (20 min)	/	/	/	/	1time/week
5–8	LI-BFRT	Seated biceps curl, Seated shoulder press Seated knee extension, Squat	4	12, 10, 10, 10	30s	Phase 2 25%1RM	3times/week
	rTMS + LI-BFRT	M1 area, at 10 Hz, 90%RMT, intermittent stimulation, a total of 2,000 pulses; LI-BFRT immediately after the stimulation ends					
	CG	Education session (20 min)	/	/	/	/	1time/week
8–12	LI-BFRT	Seated biceps curl, Seated shoulder press Seated knee extension, Squat	4	12, 10, 10, 10	30s	Phase 3 30%1RM	3times/week
	rTMS + LI-BFRT	M1 area, at 10 Hz, 90%RMT, intermittent stimulation, a total of 2,000 pulses; LI-BFRT immediately after the stimulation ends					
	CG	Education session (20 min)	/	/	/	/	1 time/week
M1 area: primary motor cortex							

#### 2.5.1 LI-BFRT

Participants will undergo a 12-week intervention consisting of 3 sessions per week, each lasting 40 min, using LI-BFRT (with dumbbells and leg sandbags as resistance load forms). The 40-min intervention includes a 5-min warm-up under the guidance of an instructor, a 30-min formal intervention, and a 5-min cool-down. According to the study by Cook et al. (37), the load is set at 20%–30% of the estimated 1RM, with 4 sets (12-10-10-10), 30 s of rest between sets, and 1 min of rest between training intervals. The four resistance exercises included two upper-body exercises: seated biceps curl and seated shoulder press; and two lower-body exercises: seated knee extension and squat.

The estimated 1RM is calculated using the maximum number of repetitions and the second-maximum load to determine the load for resistance training. First, the subject is given an appropriate load and asked to perform as many repetitions as possible. If they can perform more than 10 repetitions, they rest for 3 min and then attempt the same exercise with a heavier weight. When the maximum number of repetitions is 10 or fewer, the load is considered the second-maximum load. The formula for estimating 1RM is as follows (38):

$$\begin{array}{rl} 1\mathrm{RM}\; & =\;\mathrm{Submaximal}\;\mathrm{load}\;(\mathrm{kg})/(102.78-2.78\\ & \;\times \;\mathrm{Maximum}\;\mathrm{number}\;\mathrm{of}\;\mathrm{repetitions})/100 \end{array}$$

During training, inflatable cuffs (Theratools®, Germany) are placed on the proximal limbs, and individualized AOP will be determined before the intervention begins. A Doppler ultrasound imaging diagnostic device was used to detect the AOP of the upper and lower limbs. The compression cuffs were placed on the proximal parts of the participants' upper arms and thighs. Subsequently, the ultrasound probe was placed on the radial artery of the upper limb and the tibial artery of the lower limb. The cuff was inflated until the arterial pulse disappeared and no arterial blood flow was detected by the ultrasound probe, and the pressure at this time was the AOP. The compression intensity of the cuff was set at 60% of the AOP (39).

#### 2.5.2 rTMS+LI-BFRT

Participants will undergo a 12-week intervention consisting of three sessions per week, each lasting 60 min, combining rTMS (20 min) and LI-BFRT (40 min). The rTMS protocol is as follows: stimulation intensity of 90% of the RMT, is applied to the primary motor cortex (M1) with a stimulation frequency of 10 Hz, each sequence lasting 10 s, and an interval of 50 s. A total of 1,000 pulses are administered to each hemisphere, resulting in a total of 2,000 pulses for the rTMS intervention. Immediately thereafter, the same exercise intervention as the LI-BFRT group was performed.

The RMT will be determined before the start of the intervention. Measurements and interventions were performed using a MagTD transcranial magnetic stimulator (Irid Medical Equipment New Technology Co., Ltd., Wuhan, China) equipped with an aimQ tracking navigation system. Signals will be amplified and band-pass filtered between 20 Hz and 1 kHz, with a sensitivity of 200 μV/div and a sampling rate of 5 kHz. Subjects were instructed to maintain complete relaxation of the target muscle during stimulation and confirm the absence of background activity via real-time EMG feedback. First, activate the MEPs monitoring module. Surface electromyography (EMG) of the abductor pollicis brevis muscle is recorded using dedicated electrode wires. Align the center of the “8-shaped” coil with the representative region of the cerebral cortex associated with the hand function, ensuring the coil is tangent to the skull surface at a 45° angle. Subsequently, sTMS to observe reactive movements in the affected fingers. The RMT is defined as the minimum magnetic stimulation intensity required to elicit MEPs greater than 50 μV in at least 5 out of 10 consecutive sTMS stimuli while the thenar muscle is in a relaxed state.

#### 2.5.3 Control group

Participants in the control group will not undergo any exercise intervention and will maintain their original lifestyle habits. However, they will participate in a 12-week health education course, once a week for 20 min per session, to learn about skeletal muscle and exercise health.

### 2.6 Outcome assessment

#### 2.6.1 Primary outcome

##### 2.6.1.1 Muscle cross-sectional area (CSA)

CSA is diagnosed by an experienced radiologist. Lower limb muscle cross-sectional area is measured using a CT spiral scanner (uCT520, United Imaging Medical Technology Co., Ltd, Shanghai, China). Patients are instructed to lie in a supine position, ensuring that the CT scan plane is perpendicular to the coronal plane of the lower limbs. The scanning regions include: the thigh muscle cross-sectional area, the midpoint of the line connecting the greater trochanter of the femur and the lateral condyle of the femur (40), the calf muscle cross-sectional area, the midpoint and the upper third of the line connecting the inferior border of the patella and the lateral malleolus (41).

##### 2.6.1.2 Muscle strength

Grip strength, which represents upper limb strength, was measured using a handheld grip dynamometer (JAMAR smart, USA). Participants sat with their elbows flexed at 90° and their upper limbs immobilized while holding the grip dynamometer. Upon receiving the “start” command, they gripped the inner and outer handles with maximum force, and the peak grip strength was recorded. Three measurements were taken for each hand. There was a one-minute interval between each measurement, and the final result was the maximum value of the three measurements. Measure the maximal voluntary contraction (MVC) of knee extension of the lower limbs using a portable muscle strength tester, micro FET3 (Hoggan Health Industries, USA). During the test, the subject sat on a chair or bed with both feet suspended in the air, the hip and knee joints flexed at 90°, and the tester held the device in front of the subject's lower leg near the ankle joint. Upon the “start” command, the participant performed knee extension with maximum force, while the tester applied resistance using the portable muscle strength tester micro FET3 for 4 s, recording the peak muscle strength.

#### 2.6.2 Secondary outcomes

##### 2.6.2.1 Body composition

Body composition was measured using a body composition analyzer (Inbody S10, Korea). Participants should remain normally hydrated before testing. After cleaning their hands and feet, participants stand on the device's electrode plate, ensuring that both feet are precisely aligned with the electrodes, with the heels placed on the origin mark. Participants are instructed to hold the handle with their thumbs touching the oval electrode pads and their four fingers wrapped around the handle, with both arms extended at a 45° angle and kept straight. Throughout the test, participants are required to remain still and avoid moving their limbs or talking. The BIA device will automatically measure participants' body fat, body fat percentage, lean body mass, and visceral fat.

##### 2.6.2.2 Physical performance

###### 2.6.2.2.1 5-time sit-to-stand test

Participants sit on a chair without armrests (height: 45 cm), with both feet flat on the ground, back not leaning against the chair back, and hands crossed over the chest. Upon hearing the “start” command, they perform 5 repetitions of standing up (with knees fully extended when standing) and sitting down as quickly as possible. The time taken to complete the 5 repetitions is recorded (in seconds). The test is conducted three times, with a 1-min rest interval between each trial. The average of the three test times is used as the final result.

###### 2.6.2.2.2 30-second stand test

Participants sit on a chair without armrests (height: 45 cm), with their feet flat on the ground, their backs not leaning against the backrest, and their hands crossed in front of their chests. Upon hearing the command “start,” they perform the actions of standing up (with knees fully extended) and sitting down as quickly as possible. The number of times the participant completes the actions within 30 s is recorded.

###### 2.6.2.2.3 Timed up-and-go test

Participants are required to stand up from a chair with armrests at a height of approximately 45 cm, walk in a straight line for 3 m, turn around, return, and sit down. The tester uses a stopwatch to record the total time from the start to when the participant is fully seated.

###### 2.6.2.2.4 30-second arm curl test

Participants sits on a straight-backed chair (height: 45 cm), with both feet flat on the ground, the back firmly against the chair back, and the non-test arm hanging naturally or resting lightly on the thigh. The tested arm holds a 2-kg dumbbell, with the arm fully extended and naturally hanging at the side of the body. Upon hearing the “start” command, using the elbow joint as a pivot point, quickly raise the dumbbell to shoulder height, then slowly lower it. Throughout the movement, the arm must not touch the thigh. Continue for 30 s. Record the number of correct repetitions completed.

###### 2.6.2.2.5 Cortical excitability

Cortical excitability was assessed via MEPs amplitude. Single-pulse transcranial magnetic stimulation (sTMS) was delivered to the primary motor cortex (M1) using the MagTD device at 100%–120% of each participant's resting motor threshold (RMT). MEPs were recorded from the right abductor pollicis muscle at rest, and peak-to-peak amplitudes were measured. Six consecutive pulses were applied at 5-s intervals, with the mean amplitude serving as the excitability index. To account for inter-individual variability, MEPs were normalized to baseline values. If baseline variability exceeded 15% between groups, analyses were adjusted using either stratification or ANCOVA with baseline MEPs as a covariate.

##### 2.6.2.3 Hematological parameters

Blood samples will be collected from each participant's upper arm vein by professional medical staff before baseline (Week 0) and after the intervention (Week 12). Participants will be required to fast for 12 h before blood collection. Blood will be collected using separator tubes (5 ml), allowed to stand at room temperature for 30 min, and then centrifuged in a centrifuge for 15 min to separate serum and plasma. The centrifuge will be set to 4°C and 3,000 rpm. The serum will be aliquoted into EP tubes and frozen at −80°C for analysis. Blood markers include: creatine kinase (CK), insulin-like growth factor 1 (IGF-1), myostatin, interleukin-6 (IL-6), total testosterone (TT), estradiol (E2), neurofilament light chain (NfL), and C-terminal agrin fragment (CAF).

### 2.7 Sample size estimation

Sample size estimation was performed using G*Power 3.1. Based on a previous study (42), the effect size was conservatively estimated to be 0.65, to ensure a conservative and robust study design, we adopted a more cautious effect size of 0.4 in the final calculation. Assuming a one-way ANOVA with three independent groups (1:1:1), a significance level of 0.05 (two-tailed), and a desired statistical power of 0.95, the required total sample size was determined to be 42 participants. With an attrition rate of 20%, a total of 54 participants (18 per group) needs to be recruited.

### 2.8 Statistical analysis

All data were analyzed using SPSS Statistics version 26 (SPSS Inc., USA). Continuous variables are presented as mean ± standard deviation. Baseline characteristics were compared using one-way ANOVA for normally distributed data or the independent-samples rank-sum test/chi-square test for non-normal or categorical data. Intervention effects over time were assessed using two-way repeated-measures ANOVA (time × group) across three time points (baseline, post-intervention, and long-term follow-up). Significant interactions were followed by Bonferroni-corrected pairwise comparisons. Pearson correlation was used to explore relationships between changes in cortical excitability, serum biomarkers, and muscle mass or functional outcomes. Potential synergistic effects of rTMS and LI-BFRT were further evaluated using factorial ANOVA, with significance set at *P* < 0.05.

### 2.9 Safety monitoring and data management

The study followed strict safety and data management protocols. Trained staff continuously monitored participants, provided standardized guidance, and minimized exercise-related risks. While rTMS and LI-BFRT are generally safe, mild and transient adverse events may occur, including headache, scalp discomfort, or dizziness for rTMS, and temporary vascular discomfort, numbness, or mild subcutaneous petechiae for LI-BFRT. All sessions were supervised, and any adverse events were promptly recorded and managed. Persistent or serious events led to immediate cessation of the intervention, medical evaluation, and reporting to the ethics committee within 24 h. Participants could withdraw at any time.

Data management utilizes computer systems for recording and storage. All storage devices and databases are encrypted, with personal information replaced by digital codes. Furthermore, an independent data monitoring group composed of clinical experts, researchers, trial personnel, and statisticians is established to oversee data quality and research compliance throughout the process, ensuring the scientific rigor and reliability of the study.

## 3 Discussion

This study describes a novel integrated intervention protocol that combines rTMS with LI-BFRT for the first time to address the decline in skeletal muscle mass and function in postmenopausal women. Menopause is one of the most prominent manifestations of the aging process in women, leading to ovarian dysfunction and hormonal imbalance. This can result in systemic changes in postmenopausal women, such as reduced muscle mass, decreased muscle strength, and increased obesity, which in turn increase the risk of cardiovascular disease and metabolic syndrome (14, 43). Currently, exercise intervention, especially resistance training, is considered the primary non-pharmacological treatment for controlling muscle mass and strength loss in older people (44). Resistance training can delay or reverse sarcopenia, increase skeletal muscle mass and strength, improve physical function, enhance activities of daily living, and reduce the risk of falls (20). The effectiveness of resistance training is largely dependent on the resistance load. Compared to moderate-to-low resistance training, high-load resistance training has a greater impact on increasing strength in older people (45, 46). Since the elderly often struggle to achieve the recommended exercise load (47), there is an urgent need for new effective intervention measures.

This study employs BFRT by using pneumatic cuffs to partially or completely block arterial and venous blood flow during exercise (23), enabling the achievement of effects similar to those of traditional high-intensity resistance training under low-intensity resistance loads (48), this method has been studied in some older people (49, 50). However, studies involving postmenopausal women have not been widely conducted. Therefore, it is necessary to conduct a study on LI-BFRT involving postmenopausal women to clarify the efficacy of this method for this population, such as changes in skeletal muscle, hormone levels, and other parameters, and to explore its potential mechanisms and safety.

Moreover, cerebral cortical atrophy, motor neuron apoptosis, and neuromuscular junction (NMJ) degeneration induced by aging are considered key factors in the occurrence of senile sarcopenia (29, 31). Therefore, when the effect of simple skeletal muscle training is limited, increasing the number of motor units from the perspective of activating the central nervous system may be a new approach to improving or enhancing the efficacy of exercise interventions for skeletal muscle mass and function in postmenopausal women. TMS regulates cortical plasticity through time-varying magnetic fields, inducing electric fields to stimulate target cortical regions. It has been widely used in the treatment of neurological and psychiatric disorders associated with excitatory dysfunction of the brain. Among these, rTMS is commonly used to modulate cortical excitability, which can be evaluated through changes in MEPs (33, 51). Research has shown that rTMS can significantly alter motor cortex excitability and plasticity, thereby improving limb motor function (35, 36). In sports training applications, it has also been found that high-frequency rTMS can increase motor cortex excitability (52), while low-frequency rTMS can decrease motor cortex excitability (53). However, there are no reported applications for interventions targeting individuals with reduced skeletal muscle mass and strength or sarcopenia, particularly postmenopausal women.

This study has several advantages. First, this study is the first to combine non-invasive brain stimulation with BFRT in postmenopausal women, using a dual-target strategy aimed at simultaneously activating central (motor cortex) and peripheral (musculoskeletal) pathways to promote muscle hypertrophy and strength in older women under low mechanical stress conditions. This provides new insights for healthy interventions targeting skeletal muscle in older women. Second, this study comprehensively assessed outcomes related to muscle structure (muscle CSA) and function (muscle strength, physical activity function) in postmenopausal women, as well as cortical excitability and blood-based neuromuscular biomarkers. This design facilitates the evaluation of intervention effects from multiple dimensions and may provide potential mechanistic insights into the interactions between neural plasticity and muscle adaptability in mixed intervention responses.

Despite the novelty of this protocol, it still has some limitations. First, individual differences in response to rTMS may influence study outcomes. Second, rTMS and BFRT may require trained professionals and specialized equipment, which may limit the protocol's scalability in community settings. Finally, the study lacks advanced neuroimaging techniques, which limits the ability to deeply interpret the mechanisms of the intervention. Future research will aim to optimize these limitations and strive to translate this protocol into broader clinical practice.

## Data Availability

The datasets generated during the present study will be available from the corresponding author on reasonable request.
